# Malignant Intracerebral Nerve Sheath Tumors: A Case Report with Review of the Literature

**DOI:** 10.1155/2013/384076

**Published:** 2013-09-26

**Authors:** Faris Shweikeh, Doniel Drazin, Sergei I. Bannykh

**Affiliations:** ^1^Department of Neurosurgery, Cedars-Sinai Medical Center, Maxine Dunitz Neurosurgical Institute, Los Angeles, CA 90048, USA; ^2^Department of Pathology and Laboratory Medicine, Cedars-Sinai Medical Center, Los Angeles, CA 90048, USA

## Abstract

The occurrence of benign nerve sheath tumors within the neuroaxis is uncommon. Even rarer is the finding within brain parenchyma, termed malignant intracerebral nerve sheath tumors (MINST). We present a case of MINST which occurred in the frontal lobe of an 18-year-old male that recurred almost 4 years later. Imaging demonstrated a 4.0 cm lesion with an associated mass effect. He underwent a right fronto-parietal craniotomy for gross total resection. Pathology was inconclusive with a Glioblastoma Multiforme (GBM) as the most likely diagnosis, though gliosarcoma and MINST were also highly considered. Postoperatively, he was treated with chemotherapy and radiation and followed for almost 4 years, when an MRI indicated a recurrence. Resection of the recurrence was highly suggestive of MINST. Surgery was followed by radiation and chemotherapy, but, less than 7 months later, he was readmitted for a surgical-site infection, and, after multiple surgeries, and his family terminated care. Recognizing this unusual tumor in the differential diagnosis of a heterogeneously enhancing intracerebral mass can help surgeons diagnose and treat it. This report also exhaustively reviews the literature and presents diagnostic and treatment strategies.

## 1. Introduction

Malignant peripheral nerve sheath tumors (MPNST) typically originate from nerves of the extremities and trunk or from preexisting neurofibromas. While being very uncommon in the general population, the lifetime incidence of this neoplasm in patients with neurofibromatosis type 1 (NF1) is estimated at 8%–13% [1].

MPNST that arise from brain parenchyma are termed malignant intracerebral nerve sheath tumors (MINST) and are exceptionally rare with only 15 documented cases in the literature (Table 1). MINST has replaced the term “malignant schwannoma” because schwannomas do not have a tendency towards malignant transformation [2, 3]. Among the documented cases, a variant with rhabdomyoblastic differentiation, referred to as malignant triton tumor, has been reported as well [4–6]. 

In addition to NF1 patients, others predisposed to MINST include those with a history of ionizing radiation exposure [7, 8]. The majority of MINST cases are sporadic and have afflicted patients as young as 1 year and as old as 75 years. Not surprisingly, the diagnosis of these tumors may be problematic, and the obscurity of their cellular origin further complicates matters [2, 3, 9]. Treatment almost always involves surgery. While most patients exhibit an uneventful postoperative recovery, long-term outcomes are variable. Even with multimodality treatment, many cases are plagued with recurrences and terminal results. 

We present the unique case of a frontal lobe MINST which mimicked a Glioblastoma Multiforme (GBM) both radiographically and histologically. Only 15 cases of MINST have been reported in the literature. This case is the eighth in a child, the fifth associated with NF1, and the first masquerading as a GBM. We also present a review of the literature with a summary of factors including tumor size, location, and histological grade.

## 2. Case Presentation

### 2.1. History

Approximately 4.4 years ago, an 18-year-old right-handed male presented to the emergency department complaining of awakening in the middle of the night with left side numbness (arm, leg, and side of the face) and severe headache. The pain was refractory to over-the-counter medication and worsened throughout the morning with associated vomiting. The left-sided numbness also did not diminish and was accompanied by pain within the left upper extremity. He had been diagnosed at age 9 with an optic glioma and treated with one year of temozolomide chemotherapy and radiation. His past medical history was also significant for NF1, ADHD, Tourette's syndrome, shingles, juvenile rheumatoid arthritis, and depression.

### 2.2. Examination

**Figure 1:**
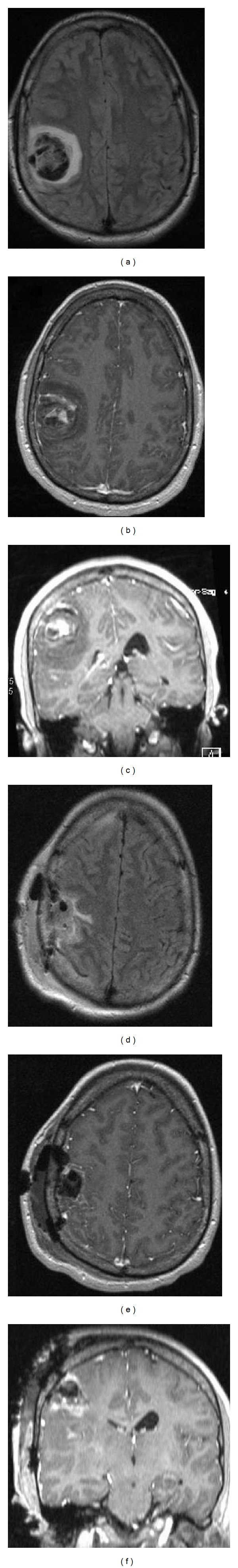
Preoperative axial flair MRI (a) and axial/coronal T1 MRI ((b),(c)) showing a large hemorrhagic infiltrative mass at the right fronto-parietal region. Postoperative axial flair MRI (d) and axial/coronal T1 MRI ((e),(f)) showing a 1 cm persistent tumor within the surgical cavity.

The neurological exam was significant for a light left upper extremity pronator drift, 4/5 strength in the left upper and lower extremities, and decreased sensation to light touch and pinprick in his left face, arm, and leg. CT scan without contrast revealed an acute parenchymal hematoma in the right fronto-parietal region with a predominantly central high density and areas of low density, which may have represented active bleeding. The area measured 4.0 cm maximum dimension with a mild amount of surrounding vasogenic edema. MRI with contrast revealed a hematoma measuring 4.1 cm AP × 3.5 cm transverse × 4.1 cm craniocaudal with an associated mass effect resulting in approximately 5 mm leftward midline shift (Figure 1). Centrally within this mass, there were nodular areas of enhancement.

### 2.3. Operation

The patient underwent a right fronto-parietal craniotomy for resection of what was suspected to be a hemorrhagic right posterior frontal glioma. A generous right fronto-parietal craniotomy was done, and the dura was dissected free and opened in a cruciate fashion. Active arterial bleeding was encountered on the cortical surface from abnormal mass. The exact location and dimensions of the hemorrhagic mass were delineated with intraoperative ultrasound. With the help of the surgical microscope, the hematoma cavity was entered and then evacuated using gentle suction and irrigation. Multiple biopsies and frozen sections were reviewed by a pathologist and were suggestive of GBM. Using standard microsurgical techniques, an anatomical plane between the hemorrhage/hemorrhagic mass and the gliotic brain was developed. The hemorrhagic mass was removed in a piecemeal fashion. After complete resection was attained, immaculate hemostasis was performed and ultrasonography confirmed an excellent resection.

### 2.4. Pathology

**Figure 2:**
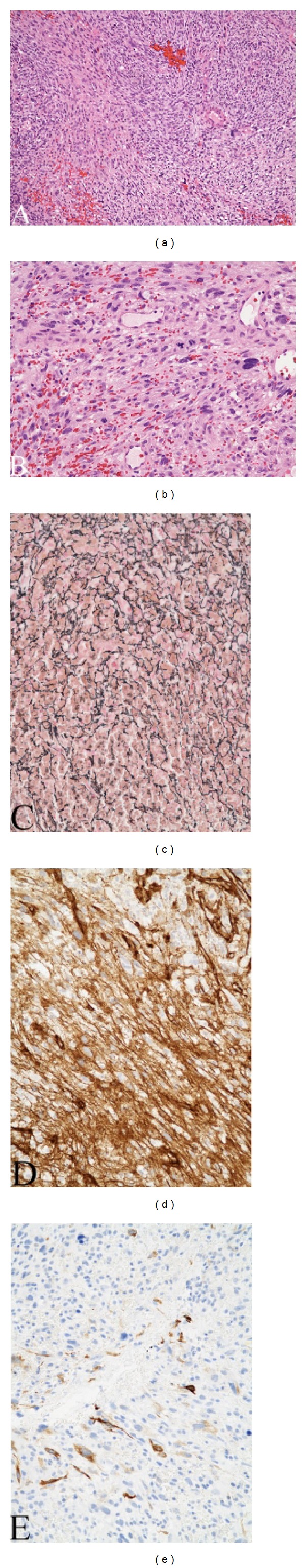
Hematoxylin and eosin (H&E) stained sections of the initial resection shows fascicular (a) to a less organized (b) arrangements of enlarged hyperchromatic nuclei with atypical mitotic figures. Cells are entrapped by a reticular network (c), containing collagen IV (d). Some of the cells are positive for smooth muscle marker desmin (e). Original magnifications: 50x (a), 200x ((b)–(e)).

**Figure 3:**
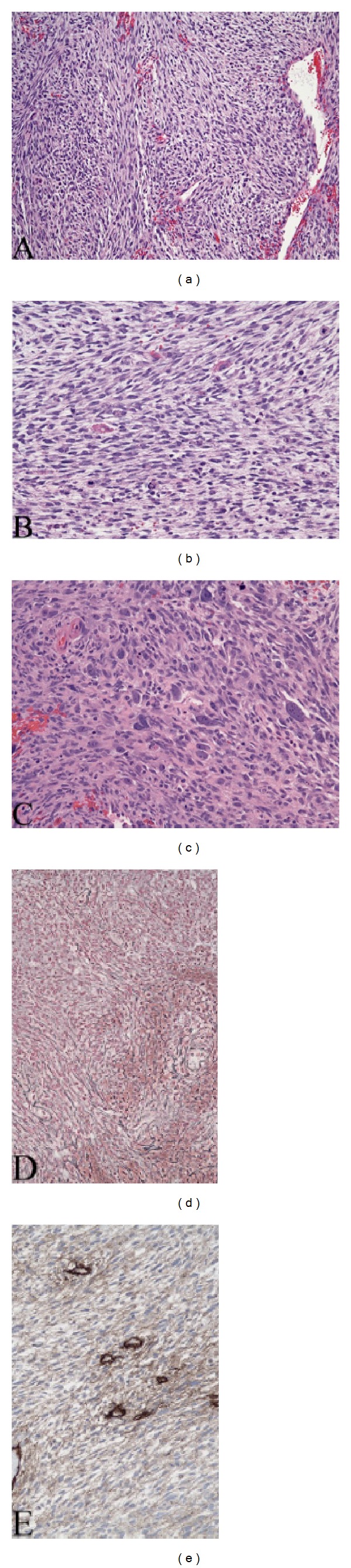
Resection of tumor recurrence showing frank sarcoma composed of intersecting fascicles spindle cells (a), often in a loose arrangements (b), or showing epithelioid changes (c). Granular cytoplasmic changes were evidence (b). Tumor cells are enmeshed by reticulin (d) and collagen IV-containing basal laminas (e). Original magnifications: 100x (a), 200x ((b)–(e)).

Grossly, the tumor was a 2.2 × 2.0 × 0.4 cm aggregate of soft, hemorrhagic, brown-tan pieces of tissue. Microscopic sections disclosed highly pleomorphic cells with hyperchromatic nuclei and numerous mitotic figures (Figure 2). Some cells exhibited vacuoles and granular changes. Foci of necrosis were present. The tumor showed a fascicular architecture reminiscent of MPNST. Reticulin staining showed focal pericellular deposition. Immunostains were negative GFAP, neurofilament, S-100, EMA, CD117, CD34, and Myo-D1. There was positivity for collagen IV in basal lamina distribution, weak positivity for desmin, and diffusely strong positivity for Vimentin. Although the initial pathological diagnosis was GBM, the subsequent panel of immunostains gathered more evidence indicating a diagnosis of a sarcoma such as MINST. Lack of S-100 positivity did not either substantiate the diagnosis of MINST or entirely exclude it. However, a recurrent tumor resected almost 4 years later showed no apparent infiltration of CNS parenchyma, as would be expected for a diffuse glioma. The second pathologic interpretation was therefore of a high-grade malignant spindle cell neoplasm, similar to the previous lesion (Figure 3). A fascicular pattern with marked anisonucleosis, nuclear hyperchromasia, mitotic figures, multinucleated giant cells, dystrophic calcification, and focal necrosis was reported. Again, the tumor cells were positive for reticulin, desmin (focal), CD10, and collagen IV (pericellular) while still negative for S-100, GFAP and also smooth muscle actin, and CD57. Electron microscopy revealed a dense cellular proliferation of intersecting fascicles, small nuclei with prominent nucleoli and frequent multinucleation, mitotic figures, poorly developed organelles, no granules or melanosomes, and no appreciable basal lamina. In short, the findings were of a poorly differentiated spindle cell neoplasm, most likely a MINST.

### 2.5. Postoperative Course

The patient awakened after surgery without any motor or sensory deficits. He was given radiation and 5 cycles of temozolomide chemotherapy. MRI demonstrated a persistent 1 cm enhancing lesion in the right frontal lobe within the surgical cavity (Figure 1). This lesion persisted and remained unchanged for the next 3.7 years.

**Figure 4:**
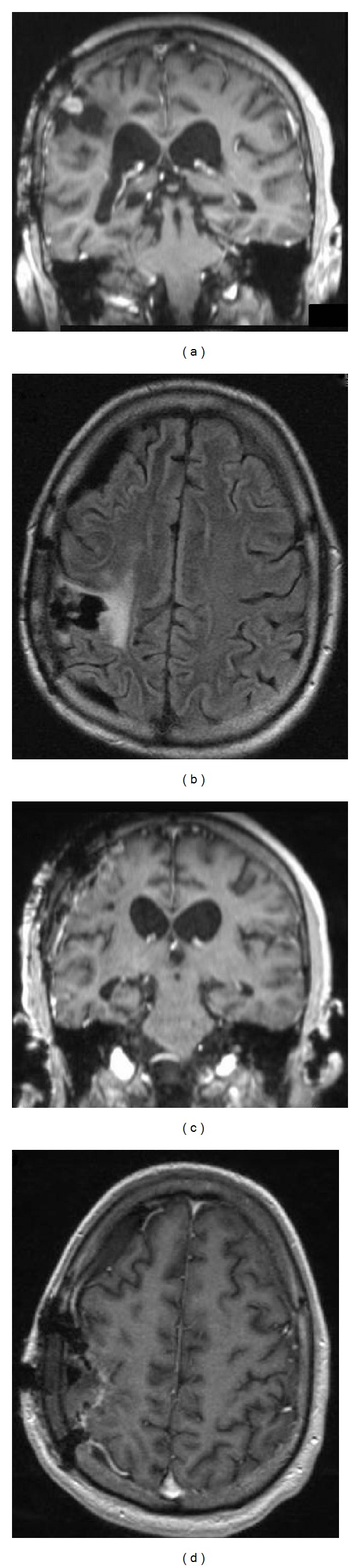
((a),(b)) Preoperative axial flair MRI and T1 coronal MRI showing a recurrent new 7 mm nodular area of enhancement at the superior aspect of the surgical cavity associated with the dura. ((c),(d)) Postoperative axial flair MRI and T1 coronal MRI showing resection of the lesion.

At 3.7 years after the initial surgery, there was evidence of a new 7 mm nodular area of enhancement at the superior aspect of the surgical cavity associated with the dura in the right frontal lobe with flare changes (Figure 4). The patient underwent surgical resection of the lesion via a right parietal craniotomy. Postoperatively, he had left-sided hemiparesis with pathology consistent with MINST. Six weeks later, the patient experienced an increased frequency of seizures. An MRI showed an enhancing nodular lesion in the resection cavity with a hyperintense fluid collection. The patient underwent a right frontal craniotomy and was found to have a reddish, round, soft tumor in the anterior superior aspect of the resection cavity with surrounding purulent-like material in the resection cavity. Cultures showed abundant *Propionibacterium acnes*, and he was treated with appropriate antibiotics. Pathological analysis of the lesion confirmed the same neoplasm. The patient was started on a rehabilitation program and received radiation with concurrent temozolomide chemotherapy. Seven months later (which is approximately 4.4 years after initial diagnosis), the patient was readmitted for an infection in the surgical site, and, after multiple operations, the family decided to withdraw care.

## 3. Discussion

Primary MINST are extremely unusual. Similar to its MPNST counterpart, the diagnosis of MINST can be formidable and necessitates a thorough investigation of clinical findings, imaging features, and histopathological characteristics. The fact that the cellular source of these tumors is uncertain further complicates matters. Some suggest an origin from Schwann cells of perivascular nerves while others favor pluripotent mesenchymal cells, but their source remains debatable [2, 3, 16]. A comprehensive histopathological analysis is warranted and includes detailed observation of cellular structure, immunostaining, and electron microscopy. Fortunately, as suggested by Barnard et al. [9], recognizing the similarity of MINST with the more common MPNST can help guide diagnosis and treatment. MPNST generally have poor outcomes because of their invasive nature and ability to metastasize to various organs [1]. Factors that influence survival include tumor size, location, and presence of NF1 [13]. Multimodality treatment is usually instigated with the aim of achieving gross total resection (GTR) followed by radiotherapy and chemotherapy, if necessary.

Table 1 summarizes the characteristics of previously reported cases of MINST. Tumor location was variable with cases reported in all major lobes of the cerebrum and a few with intraventricular and cerebellar involvement. In our case, the tumor was intraparenchymal with extension to the dura mater, a unique finding among the cases reported thus far. Overall, 4 (26.7%) cases were infratentorial. The male to female ratio was almost 1 : 1, and 7 (46.7%) of the patients were children (age 18 or younger). Clinical presentation was also wide-ranging and depended on tumor location with a number of patients presenting with signs of increased intracranial pressure. Radiographically, the tumor typically disproportionately enhances with areas of calcification and/or necrosis. The heterogeneous mass may also contain loculations or cystic formation (Table 2). While it can be indistinguishable from malignant glioma on imaging, a recent study revealed MR spectroscopy as a potential tool in differentiating glial versus nonglial cellular origin [11]. Namely, the presence of a high choline peak without creatine and N-acetyl aspartate resonance suggests a tumor of nonglial source.

In terms of cytopathological findings, our patient shared similar characteristics with previously reported MINST cases. This included hypercellular spindle cells with marked variation in cellular and nuclear size, interlacing fascicular arrangement, signs of nerve sheath differentiation (i.e., discontinuous cell junctions, intertwining cytoplasmic processes, underdeveloped external lamina, etc.), presence of syncytial epithelioid tumor cells and multinucleated giant cells, and foci of necrosis (Table 2). Though many previous cases of MINST had expression of S-100, our case did not and its presence could not confirm nor exclude the diagnosis. Malignant triton tumors had similar elements with the additional finding of a rhabdomyoblastic component [4–6]. A reasonable explanation suggested for this transformation is the ability of malignant Schwann cells to differentiate into muscle cells [4, 19]. Differential diagnosis of MINST includes gliosarcoma, gliofibroma, desmoplastic astrocytoma, meningioma, rhabdomyosarcoma, malignant solitary fibrous tumor, and gastrointestinal stromal tumor. These were ruled out by negative stains for GFAP, neurofilament, IDH-1, EMA, actin, MyoD1, CD34, and CD117. Table 2 summarizes the histopathological and radiological findings in the previously reported cases. 

Like MPNST, patients with MINST have varying outcomes with many having a poor prognosis [10, 12, 15, 17, 18] (Table 1). After tabulating results from the few reported cases, it was found that, at mean follow-up of 13.5 months, 7 (50.0%) patients had at least one recurrence and 5 (35.7%) remained alive. Sharma et al. were the first to note that the earlier the first recurrence, the worse the overall survival [14], and this is supported by the results outlined in Table 1. For almost all cases, surgery with the goal of GTR was first-line treatment. As with MPNST, achieving complete resection can improve long-term outcomes [1, 9, 20]. This can be complemented with radiotherapy and/or chemotherapy, but their effect on prognosis is inconclusive. Nine (60%) of the reported cases received radiotherapy, 3 (20%) of which also received chemotherapy. Overall survival was not any better than receiving GTR alone, although, in a case reported by Beauchesne et al., survival was 29 months following a combination of radiotherapy and chemotherapy [2]. Hence, the inherent biological tendency of MINST could differ from case to case, and it is difficult to make any conclusions on outcomes based on size, location, and histological grade. Many more case reports are required to make any conclusive generalization about this rare tumor, though it does tend to be a substantially invasive and aggressive tumor.

Our patient did well for more than 4 years with surgery along with radiation and chemotherapy. With a survival of approximately 52 months, it is the second longest survival reported in the literature. Additionally, as this is the fifth case of MINST in a patient with NF1, it adds further evidence to the possible association of NF1 with this neoplasm.

Awareness of this tumor, extensive surgical extirpation, and thorough histological examination serve as essential components in management. It is recommended that MINST be part of a differential diagnosis for a patient with clinical and radiographic signs of an intracranial tumor as MINST have the potential to mimic glioblastomas. Reports of more cases with lengthier follow-up are needed to gain a better understanding of the effect of these modalities and help guide management plans for this infrequent tumor. 

## 4. Conclusion

MINST are rare, frequently recurring, aggressive malignant tumors with infiltrative capability which can exhibit clinical and radiographic signs of an intracranial tumor and mimic a glioblastoma. Recognizing the similarity of MINST to their peripheral counterparts, MPNST, and their possible relationship with NF1 can help surgeons correctly diagnose and treat this tumor. Although arduous, the diagnosis is confirmed through comprehensive histopathological analysis. The standard of care is surgical GTR, sometimes followed by radiation therapy and/or chemotherapy, and close, frequent follow-up due to its invasive nature and high risk of recurrence. Considering this unusual tumor in a differential diagnosis of a heterogeneously enhancing intracerebral mass can help surgeons recognize cases of MINST. More case reports and studies are needed for conclusive evidence for the best approach to managing MINST and in determining factors that influence survival.

## Figures and Tables

**Table 1:** Clinical findings and outcomes in 15 previously reported cases of MINST.

Authors [reference]	Age (years)/ sex	Location	Special aspect	Clinical presentation (duration if reported)	Treatment	Recurrence (months)	Follow-up (months)	Survival (at last F/U)
Ellis et al., 2011 [7]	9/F	Right fronto-temporal	NF-1	Headaches (2 months)	STR + chemo + RT	No	6	Alive
Barnard et al., 2011 [9]	75/F	Left frontal lobe	—	Personality changes, aphasia, gait instability, and confusion	GTR + RT	No	12	Alive
Oztanir et al.,2009 [10]	1/F	Right fronto-temproparietal	NF-1	Developmental delay, vomiting, and lethargy	STR	NA	1.5	Death at 6 weeks due to sepsis
Kozić et al., 2008 [11]	39/M	Left intrapontine	—	Left facial paresthesia, ataxia, and dysarthria (12 months)	Biopsy	NR	NR	NR
De Cauwer et al., 2007 [5]	68/F	Left parieto-frontal (rolandic area)	NF-1; TV	Syncope, dyssthesia of left arm, and seizure	GTR + RT	5	5	Dead
Beauchesne et al., 2004 [2]	35/M	Right cerebral peduncle	—	Horizontal dipoplia, left hearing loss, headache, nausea, and ataxia (2 months)	Biopsy + RT + chemo	17	29	Dead
Maiuri et al., 2004 [12]	36/M	Cerebellar vermis	—	Headache, vomiting, dizziness, vision loss, and ataxia (2 months)	GTR + RT	6	8	Dead
Bornstein-Quevedo et al., 2003 [4]	3/M	Right parieto-occipital	TV	Frontal headache, vomiting, irritability, and aggressive behavior (2 months)	STR	NA	0.33	Died at 10 days due to ICH
Takahashi et al., 2000 [6]	57/M	Left lateral ventricle	NF-1	Memory disturbance, disorientation	GTR + RT + Chemo	No	4	Dead
Tanaka et al., 2000 [13]	4/F	Right parieto-occipital	—	Headache, vomiting	GTR	No	19	Alive
Sharma et al., 1998 [14]	8/F	Right temporal	—	Seizures	GTR	No	17	Alive
Jung et al., 1995 [15]	40/M	Right lateral ventricle	—	Headache, vomiting, and drowsiness	GTR + RT	8	8	Dead
Singh et al., 1993 [16]	61/F	Right cerebellar	—	Headache, vomiting, unsteady gait, and diplopia	GTR + RT	10	18	Dead
Stefanko et al., 1986 [17]	15/M	Left parieto-occipital	—	Headache, vomiting	GTR + RT + Chemo	5 and 8	9	Dead
Bruner et al., 1984 [18]	18/M	Frontal lobe	—	Syncope	GTR	24, 48, and 66	66	Alive
Present case	18/M	Right fronto-parietal	NF-1	Left side numbness, severe headache, pain in left upper extremity, and ICH	GTR, RT	44	52	Dead

GTR: gross-total resection; STR: subtotal resection; RT: radiation therapy; chemo: chemotherapy; ICH: intracerebral hemorrhage; NA: not applicable; NR: not reported; NF-1: neurofibromatosis type 1; TV: triton variant.

**Table 2:** Radiographic and histological features in 15 reported cases of MINST.

Authors [reference]	Radiological findings	Tumor size (cm)*	Histopathological findings
Ellis et al., 2011[7]	MRI: Irregular enhancing lesion	8 × 6.5 × 7	Spindle and epithelial cells, hypercellular, fascicular pattern, mitotic index >10 mitoses/hpf, interdigitating processes, tight junctions, basal lamina, +S-100, +Bcl2, +PGP 9.5, +BAF47
Barnard et al., 2011 [9]	MRI: heterogeneous mass with areas of enhancement, cystic changes, and foci of hemorrhage	6.8 (ap) × 5.0 (t) × 4.6 cm (ci)	Spindle cells, moderate cellularity and pleiomorphism, mitotically active, high nuclear : cytoplasmic ratio, fascicular pattern, microcysts, +Vimentin, +S-100
Oztanir et al., 2009 [10]	MRI: heterogeneous contrast enhancement, central necrosis, calcifications, cystic components, peritumoral edema, isointense (T1 and T2)	8 × 8 × 8	Spindle cells, fascicular pattern, geographic necrosis, abundant mitosis, +S-100, +Vimentin
Kozić et al., 2008 [11]	MRI: Ill-defined mass, inhomogenous, faint contrast enhancement, hyperintense on T2	3.0 (ap) × 3.2 (ci)	Tightly packed cells, arranged in fascicles, +S-100
De Cauwer et al., 2007 [5]	MRI: Perilesional edema, Methionine PET: a site of high metabolic activity.	NR	Spindle cells, hyperchromatic nuclei, prominent nucleoli, rhabdomatoid differentiation, high mitotic activity, necrosis, +S-100, +Desmin, +Vimentin
Beauchesne et al., 2004 [2]	MRI: a heterogeneous, peripherally enhancing, centrally necrotic mass	2.2	Spindle cells, highly cellular, nuclear polymorphism, fascicles pattern, +S-100, +Vimentin
Maiuri et al., 2004 [12]	CT: hyperdense; irregular margins, inhomogeneous contrast enhancement.	NR	Fused, pleomorphic cells, hyperchromatic nuclei, some multinucleated cells and epithelioid cells, mitotic index was 4 × 10 HPF, +S-100, +Vimentin, +Leu 7 (CD57)
Bornstein-Quevedo et al., 2003 [4]	CT: enhancing mass lesion, multilobulated	5.1 × 3.2	Spindle cells, rhabdomyoblasts, hypercellular, fascicular pattern, marked pleiomorphism, high mitotic activity (8 mitoses/10 HPF), necrosis, fragmented external lamina, thin cytoplasmic processes, occasional cell junctions, +S-100, +CD57, +desmin, +myoglobin, +Myo-D1
Takahashi et al., 2000 [6]	MRI: solid, irregularly enhancing mass; hypointense (T1), hyperintense (T2)	3.0 × 4.0 × 3.0	Spindle cells, mitotic activity, rhabdomyoblastic component, surrounding basal lamina, desmosomes, +actin,+desmin, +S-100
Tanaka et al., 2000 [13]	CT: isodense mass, intratumoral calcification, perifocal edema, irregularly shaped septa within multiloculations, MRI: considerable enhancement; hypointense (T1), mixed signal intensity (T2)	5.0 × 5.0 × 4.0	Hypercellularity, spindle cells, nuclear hyperchormatism, interlacing fascicles, cystic components, high mitotic activity (10/10 HPF), stromal calcification, +S-100, +NSE, +Vimentin, +MIB-1 (10%)
Sharma et al., 1998 [14]	MRI: mixed attenuation, disruption of the gray/white matter interface, multiple cysts	3.4 × 2.7 × 0.4	Moderate-high cellularity, moderate pleomorphic spindle cells, intertwining fascicles, 3 mitoses/10 HPF, +epitheloid cells, +S-100, +MIB-1 (2.3–7.5%), +reticulin
Jung et al., 1995 [15]	CT: cystic mass, peripheral edema, heterogeneous enhancement MRI: considerable enhancement; hypointense (T1), mixed signal intensity (T2)	5.0 × 5.0 × 6.0	Spindle cells, interlacing fascicles, frequent mitotic figures, thin cellular processes, distinct external lamina, +reticulin, +S-100, +Vimentin,
Singh et al., 1993 [16]	CT: contrast-enhancing, mixed attenuating lesion	NR	Spindle cells, +foamy/clear cells, many mitotic figures, interwoven fascicles, lobular outgrowths, prominent vessels, cellular processes, poorly developed basal lamina, +necrosis, +pseudoinclusions, +desmosomes, +S-100
Stefanko et al., 1986 [17]	CT: ring-enhancing lesion	6.2 × 6.2	Bipolar spindle cells, hyperchromatic nuclei, high cellularity, +epithelioid cells, +syncytia, +basal lamina, +cellular processes, +reticulin, +S-100,
Bruner et al., 1984 [18]	CT: mass lesion	NR	Spindle cells, rare mitoses, +collagen, +reticulin, rare basement membranes, occasional cell junctions, MNGCs, +bizzare cytolplasmic processes +S-100, +GFAP
Present case	MRI: hematoma with mass effect resulting in 5 mm midline shift. Nodular areas of enhancement centrally within this mass	4.1 cm × 3.5 cm × 4.1 cm	Pleomorphic spindle cells, hyperchromatic nuclei, fascicules, basal lamina, vacuoles and granular changes, anisonucleosis, mitotic figures, MNGC, dystrophic calcification, necrosis foci, +collagen, +desmin, +Vimentin

*Tumor size as reported on imaging or pathology; MNGC: multinucleated giant cells; NR: not reported.

## References

[1] Widemann BC (2009). Current status of sporadic and neurofibromatosis type 1-associated malignant peripheral nerve sheath tumors. *Current Oncology Reports*.

[2] Beauchesne P, Mosnier J-F, Schmitt T (2004). Malignant nerve sheath tumor of the right cerebral peduncle: case report. *Neurosurgery*.

[3] Hirose T, Sumitomo M, Kudo E (1989). Malignant peripheral nerve sheath tumor (MPNST) showing perineurial cell differentiation. *American Journal of Surgical Pathology*.

[4] Bornstein-Quevedo L, Peralta-Olvera F, Marhx-Bracho A, Rodríguez-Jurado R, De Leon-bojorge B (2003). Cerebral malignant nerve sheath tumor, triton tumor variant: case report. *Pediatric and Developmental Pathology*.

[5] De Cauwer H, Bogers J-P, Duwel V, Van Den Hauwe VDH, Croese P, Van Marck E (2007). An intracerebral intraparenchymatous triton tumor in a man with neurofibromatosis. *Journal of Neurology*.

[6] Takahashi Y, Sugita Y, Abe T, Yuge T, Tokutomi T, Shigemori M (2000). Intraventricular malignant triton tumour. *Acta Neurochirurgica*.

[7] Ellis MJ, Cheshier S, Sharma S (2011). Intracerebral malignant peripheral nerve sheath tumor in a child with neurofibromatosis type 1 and middle cerebral artery aneurysm treated with endovascular coil embolization: case report. *Journal of Neurosurgery*.

[8] Evans DGR, Baser ME, McGaughran J, Sharif S, Howard E, Moran A (2002). Malignant peripheral nerve sheath tumours in neurofibromatosis. *Journal of Medical Genetics*.

[9] Barnard ZR, Agarwalla PK, Jeyaretna DS (2011). Sporadic primary malignant intracerebral nerve sheath tumors: case report and literature review. *Journal of Neuro-Oncology*.

[10] Oztanir N, Emmez H, Aytar MH, Dogan M, Kaymaz M, Baykaner MK (2009). Malignant intracerebral giant nerve sheath tumor in a 14-month-old girl with neurofibromatosis type 1: a case report. *Child’s Nervous System*.

[11] Kozić D, Nagulić M, Samardzić M, Ostojić J, Rasulić L, Cvetković-Dozić D (2008). Intrapontine malignant nerve sheath tumor: MRI and MRS features. *Acta Neurologica Belgica*.

[12] Maiuri F, Colella G, D’Acunzi G, De Caro MDB (2004). Malignant intracerebellar schwannoma. *Journal of Neuro-Oncology*.

[13] Tanaka M, Shibui S, Nomura K, Nakanishi Y, Hasegawa T, Hirose T (2000). Malignant intracerebral nerve sheath tumor with intratumoral calcification. Case report. *Journal of Neurosurgery*.

[14] Sharma S, Abbott RI, Zagzag D (1998). Malignant intracerebral nerve sheath tumor: a case report and review of the literature. *Cancer*.

[15] Jung J-M, Shin H-Y, Chi JG, In Sung Park ISP, Eun Sang Kim ESK, Jong Woo Han JWH (1995). Malignant intraventricular schwannoma. Case report. *Journal of Neurosurgery*.

[16] Singh RVP, Suys S, Campbell DA, Broome JC (1993). Malignant schwannoma of the cerebellum: case report. *Surgical Neurology*.

[17] Stefanko SZ, Vuzevski VD, Maas AIR, Van Vroonhoven CCJ (1986). Intracerebral malignant schwannoma. *Acta Neuropathologica*.

[18] Bruner JM, Humphreys JH, Armstrong DL (1984). Immunocytochemistry of recurring intracerebral nerve sheath tumor. *Journal of Neuropathology & Experimental Neurology*.

[19] Woodruff J, Ortiz-Hidalgo C (1993). Origin of triton tumor. *American Journal of Dermatopathology*.

[20] Wong WW, Hirose T, Scheithauer BW, Schild SE, Gunderson LL (1998). Malignant peripheral nerve sheath tumor: analysis of treatment outcome. *International Journal of Radiation Oncology Biology Physics*.

